# Health Issues with Learning to Use Smart Devices in the Digital Age: Using a Grounded Theory Approach

**DOI:** 10.3390/ijerph18137062

**Published:** 2021-07-01

**Authors:** Myung-Sill Chung, GyeongAe Seomun

**Affiliations:** 1 College of Nursing, Seongshin University, 2 Bomun-ro, 34da-gil, Seongbuk-gu, Seoul 02844, Korea; cmsill@sungshin.ac.kr; 2 College of Nursing, Korea University, Seoul 02841, Korea; 3 BK21FOUR R&E Center for Learning Health Systems, Korea University, 145 Anam-ro, Seongbuk-gu, Seoul 02841, Korea

**Keywords:** smart learning, digital, health, grounded theory

## Abstract

The Korean government has announced a plan for a national policy to deliver smart education among all students. As a result, many people are worried about the possible health-related adverse effects. This qualitative study aimed at analyzing health issues related to middle school students who learn by using smart devices using the grounded theory approach. We conducted in-depth interviews with 30 students at four middle schools who used smart devices for more than a year. The analysis of this research data was based on the constant comparative method, using grounded theory as a theoretical framework. The data analysis revealed many concepts in 28 subcategories and 13 categories related to smart learning health issues, with the central phenomenon being experience with health problems. Students’ health-related experiences were classified as physical or psychosocial symptoms. Adverse health effects related to smart learning were related to unsafe smart learning behavior and an inefficient smart learning environment. The consequences appeared to be the formation of diverse digital habits through the ambivalent use of smart devices and differences in ability to control health problems. Our findings suggest that students can form ideal habits for using smart devices if their health issues are well monitored and managed.

## 1. Introduction

New forms of educational media have become widespread, enabling classes and learning activities through wireless communication technology without restrictions on time or place. Smart learning is a concept that combines smart devices (smart phones, tablet PCs, and new inner technologies) and is a learner-centered customized learning method. Additionally, certain learning methods use advanced information and communication technologies, as well as network resources based on such technologies [1]. Koper (2014) defines a smart learning environment as a physical environment enhanced by digital, context-aware, and adaptive devices that facilitate faster and better learning [2]. Palau (2019) stated that technology-enhanced learning makes the process of learning more effective, efficient, and enjoyable. It has also been said smart learning environments can create richer physical environments to use information or skills such as communication in classrooms [3].

The definition of smart learning encompasses different educational methods using smart devices in a broad sense. In Korea, the concept of smart learning is most commonly used by the Ministry of Education, who in 2011 created the “SMART” concept, which is defined as learning information technology involving self-directed, interest-inducing, and abundant materials to suit learners’ level and aptitude [1]. As a way to increase educational satisfaction, reduce dependence on private education, and improve classroom classes, the introduction of smart learning in Korean public education began in earnest 2011 with the establishment of the “smart education promotion strategy”. During this process, the government focused on laying the foundation for smart education (such as infrastructure construction and digital textbook development) [4].

The Korean government promotes smart learning by taking advantage of its position as a leading country in information technology [5]. An example of this form of media is smart learning. Smart learning is an educational service that relies on high-speed communication technology in a narrow sense, utilizing the advantages of smart devices such as mobility and intelligence [4,6,7,8].

During the seventh curriculum, the Korean government expanded computer education for students in elementary and middle schools, as computer education became mandatory due to increased PC communication and Internet use following the development of the IT industry [9]. As a result, the Korean government supplied digital textbooks to some elementary and middle schools as part of the pilot project. Digital textbooks are attractive teaching media with the benefits of reducing the time constraints and information gaps that exist with textbooks. This represents an innovation in education by providing students with the convenience of carrying only one tablet laptop instead of heavy school bags and various contents in multimedia format [5]. This change in the educational environment has become an important issue for growing children, as computer use has become more common not only in schools, but also in homes.

Children who were born in the digital era think of the Internet and digital devices as part of their natural environment and are called “digital natives” [10]. It is natural for digital natives to apply and utilize smart learning over the course of their education [6,7,11,12,13]; however, it is necessary to carefully examine the potential health side effects of smart learning before state-of-the-art technology is applied in the field of education [8,14]. In particular, the biggest concern for teenagers is health problems that can result from increased use of computers and smartphones [6,8,15].

Among school-age children, the use of smart devices such as smartphones is the highest for middle school students, followed by college students and high school students [16,17]. A large national survey found that middle school students [17] were the most vulnerable to smart device use. Therefore, it is clear that research on health issues related to middle school students regarding their use of smart devices is urgently needed.

Research related to smart learning is focused primarily on developing educational content and methods [5], determining educational uses [4,18], and developing the ICT capabilities of users such as teachers, students, and parents [19,20,21,22]; however, in order to form healthy habits when using computers, the focus should be on preventing health problems in advance, rather than trying to solve them later. In addition, various interventions and strategies to achieve these goals should be developed and used properly [23]. Nevertheless, there are only a few in-depth studies on potential health problems for students using smart learning in schools [5].

The most common health problem in adolescents is called visual display terminal syndrome. VDT syndrome is a term that refers to all symptoms that occur after working for a long time while watching a computer monitor, such as musculoskeletal, skin, and psychological symptoms [4,24,25]. Considered alongside these physical and psychological symptoms, interpersonal building and communication skills are also important developmental tasks for growing children. Today’s teenagers are more likely to be unable to achieve normal developmental tasks due to the excessive use of smart devices [6,7,26].

If digital learning is uniformly introduced or neglected as a matter for individuals and families with students without the verification of health problems at home or in educational environments, then various social problems are expected. In particular, for school-aged teenagers, there are many opportunities to come into contact with smart devices in their daily lives; thus, health-related issues should be prioritized above all in terms of growth [27], although the effects of the excessive use of smart devices on health cannot be determined simply based on prejudiced views, meaning fundamental findings based on objective data are needed rather than vague assumptions [28].

Qualitative research methods are appropriate for in-depth investigation on students’ potential health problems, and among them grounded theory is useful for understanding how digital learning can cause health problems by assessing student behaviors, attitudes, and habits in order to see how these problems can be solved. Grounded theory is a qualitative research method that generates theories in empirical investigations. The term “theory” here means a framework that systematically connects multiple concepts (categories) extracted from data, in turn creating analytical concepts from the data and correlating the formed concepts to organize them around one core category. Grounded theory must be theorized through analysis, so is appropriate when it is based on data [29,30]

As such, in order to understand the health-related experiences of middle school students who uses smart devices and to establish some countermeasures, it is necessary to conduct grounded theory research. Grounded theory can explore the health-related experiences of students in digital learning from the perspective of symbolic interactionism, allowing suggestions for in-depth and advanced theories.

## 2. Methods

The research design used in this study was Strauss and Corbin’s [29] grounded theory. The purpose of this design was to understand the various behaviors of middle school students on health issues experienced during smart learning.

The grounded theory method involved codes were developed into categories, which were constantly compared to other codes, with notes also being added. Memos were constantly compared and classified and data were conceptualized as the ultimate goal of the developing theories.

### 2.1. Setting and Participants

Students from 4 middle schools who had experience using smart devices for more than a year were selected as participants with the approval of their principals. A total of 30 students aged 13 to 15 years were selected for the final interview, and counseling rooms at each school were used to make sure they felt comfortable. Researchers conducted the interview by using the in-depth questionnaire shown in Table 1. In addition, the data were analyzed simultaneously to derive various concepts and categories, and based on the theoretically relevant concepts, theoretical sampling could continue. The interview ended when it was determined that theoretical saturation was reached, which was the point at which the content of the interview was repeated and no new categories appeared.

### 2.2. Ethical Considerations

The study was conducted according to the guidelines of the Declaration of Helsinki, and was approved by the Institutional Review Board of KUIRB-2021-0139-01.

### 2.3. Data Collection

The secondary data in this study were intended to give middle school students living in digital learning environments an in-depth understanding of how to solve their health-related problems. To understand how children and adolescents in the era of digital learning comprehend their health experience (KUIRB-14-24-A-1), these data were based on interviews with middle school students. In-depth interviews were conducted from 9 November to 7 December 2014. Cooperation for participant selection was obtained after explaining the background and purpose of the study to principals and parents at the target schools. After explaining the purpose and methods of the study in which interview materials would be used to students and their guardians, signed consent forms were collected. Each student participated in 1 to 3 interviews, with each one lasting about 50 min. All 30 students were interviewed using the questions shown in Table 1, but due to uncertain answers, two of them had to be re-interviewed three times for confirmation. The criteria for selection were being a middle school student with more than a year of learning experience using smart devices. We used purposive sampling. All students were asked to speak freely about their experiences. The school counseling office was used as the venue to make sure all interviews would proceed in a relaxed atmosphere without interruption. While avoiding prejudice and not reflecting it during the interview, the investigator avoided structured questions, trying to create an atmosphere that would allow participants to feel comfortable and talk freely about their experiences. The interview questions were developed by researchers. To develop the interview questions, we used the literature review of prior studies on “potential adverse health related to digital learning”. During the in-depth interviews with middle school students, their native languages were used when asking the questions. The interviewer took account of the topics according to the participant’s conversational patterns based on the questions in Table 1. The interviews were recorded with the participants’ consent; each recording was transcribed in its entirety after the interview and the transcriptions were reviewed and compared to the recordings. The research questions were as follows: Have you ever experienced any kind of health-related inconvenience or concern over the past year while using smart devices for learning? If so, how did you deal with it? The questions in Table 1 are semi-structured questions that were commonly asked of the subjects; however, some of the 30 students were asked in more depth about how they have coped with the central phenomenon of this study during the second and third interviews.

### 2.4. Data Analysis

The analysis of this research data was based on a method created by Corbin and Strauss [29]. The data analysis used the coding of “open, axial, selective” according to the grounded theory method. First, the health problems of students who experienced smart learning were meaningfully identified by dismantling and reviewing the data collected through open coding in detail to form concepts and to categorize them. Second, we worked on linking categories created by open coding through axis coding. For the central category, different categories (i.e., causal conditions, contextual conditions, interventional conditions, action–interaction, and consequences) were associated, and the paradigm model was established. In addition, the central phenomenon was linked to action–interaction to examine the changes over time. Third, the core categories were identified through selection coding and hypotheses and theories were generated.

## 3. Results

### 3.1. Open Coding

The analysis was focused on health problems that occur during smart learning. The categories of health issues related to smart learning were identified by using the grounded theory approach, as shown in Table 2.

### 3.2. Axial Coding

**Figure 1:**
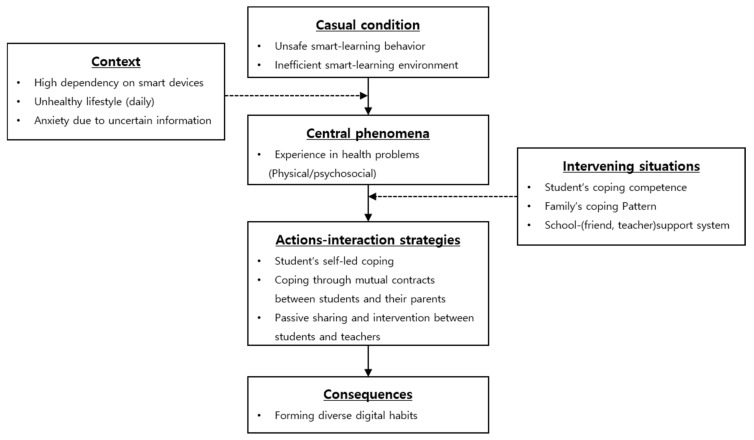
Paradigm model of health-related experiences with smart learning.

We worked on linking categories created by open coding through axis coding. In the central category, different categories (causal conditions, contextual conditions, interventional conditions, action–interaction, consequences) were associated and the paradigm model was established (Figure 1). In addition, the central phenomenon were linked to the action–interaction to examine the changes over time.

#### 3.2.1. Paradigm

##### Causal Conditions

*Unsafe smart learning behavior*: Students were found to be engaged in unsafe smart learning behavior. First, the act of listening to lectures on a smart device for a long time without any breaks was discovered. Students listened for a minimum of 2 h for 2–3 learning sessions without a break. Second, students watched lectures on their smart devices or listened to lectures while walking or riding a bus with their earphones on. Third, two notable problems were observed with regard to smart learning being better with smartphones rather than tablet PCs, because it is typical for students to focus on small letters on small screens. The distance between the eyes and the screen is closer than when using a textbook or computer; some students even said that rather than using their smartphone sitting on a chair, they prefer lying on the bed or the floor.

“My eyes hurt more when I watch lectures on a smartphone because the screen is small, and the letters are small, too. […] I hold the screen as close to my eyes as possible.”

“I think my eyes hurt because I look at the smartphone screen on a shaking bus.”

*Inefficient smart learning environment*: Students reported that they cannot concentrate due to health problems such as lower back pain because they sit on chairs with no backrest, while the computer makes it worse when it malfunctions or lags.

“My lower back hurts when I watch lectures while sitting on a chair without a backrest.”

“When loading is slow, I get annoyed. I do something else while waiting and I’m not able to study. On such days, I cannot study the lessons.”

##### Context

*High dependency on smart devices*: Students indiscriminately reported health problems on purpose because they were caused by the use of smart devices, even when they used the devices for purposes other than learning. In addition, the frequency of use was higher on weekdays when both parents were present.

“Regardless of whether I use the smartphone for study or not, my mom scolds me for using it all day long.”

*Unhealthy lifestyle(daily)*: Students who wear circle lenses (colored contact lenses worn for appearance) or use headphones instead of speakers during smart learning complained about dry eyes and uncomfortable ears.

“When I perform smart learning while wearing circle lenses, my eyes feel dry after 30 to 40 min.”

*Anxiety due to uncertain information*: In some cases, students had vague fears about negative effects related to the use of smart devices, such as exposure to electromagnetic waves and suppression of brain development, as well as other health problems they heard about in mass media or were passed down through rumors.

“The media and people around me say using a smartphone is bad for my health because of electromagnetic waves. Once I heard these claims, I became worried about the safety of smart devices.”

##### Central Phenomena

*Experience of physical health problems*: Physical health problems experienced during smart learning include the following: (1) Eye symptoms: As the eyes become easily tired, vision will degrade, and the eyes will feel heavy, stuffy, and dry. (2) Students stated that they experienced mild musculoskeletal discomfort due to moderate pain in the neck, shoulders, back, and wrists. (3) Students have impaired hearing. (4) Students describe systematic symptoms such as increased sleepiness with languor, fatigue, and dizziness.

“When I listen to a lecture with my earphones on for more than an hour, my hearing feels weaker and I feel slight pain, so I take them out. I was almost hit by a motorcycle because I was listening while I was walking.”

*Experience of psychosocial health problems*: Psychosocial health problems caused by smart learning include having a passive attitude and poor concentration for learning. When students use smart learning, they are passive about their studying method; they tend to spend more time procrastinating with Internet searches and chatting on social networks than reading textbooks.

“I just feel sleepy. […] Whenever I listen to something in a lecture that I already know, I stare blankly at the screen while thinking about something else.”

##### Intervening Conditions

*Student’s coping competence*: Subcategories of coping competence were represented by searching for and collecting health-related knowledge using digital devices. The desire to continue smart learning is referred to as a positive attitude, and solving health problems and making demands by oneself is referred to as performance ability.

“I searched for eye-massage method’ on the Internet.”

*Family coping patterns*: There are two types of family coping patterns, namely the “green” support type, which involves exchanging friendly support and sharing opinions related to health issues when using smart devices, and the “opposition” type (not limited to oneself), which emphasizes unconditional self-control regarding the use of smart devices.

“I didn’t tell my mom when my eyes hurt because I did not want to hear her nagging me that I use my computer too much. When I talked to my friend, he understood, saying he experienced the same thing.”

*School (friends, teachers) support system*: Students had the experience of sharing information and empathizing with friends about the physical discomforts caused by smart learning. In addition, teachers provided information about how to learn using smart devices with proper habits during classes.

“I think I once had a lesson about computer-use habits in health class when I was in elementary school.”

##### Action–Interaction Strategy

*Student’s self-led coping*: When students experienced health problems, some did not seek outside support. They endured the discomfort and continued to use the devices or tried to alleviate the discomfort on their own.

“When my eyes hurt while I work on the computer, I just take a break. Then the symptom goes away, and I don’t have to tell mom about it.”

*Coping through a mutual contract between studentsand parents*: Students and parents have created rules at home and set time limits for using smart devices, and attempt to abide by the rules. They have also adopted various tools such as backrests and monitor stands to reduce discomfort.

“My mom takes my cellphone away after nine o’clock in the evening on weekdays. And my computer use is limited, meaning I can only use the computer when my parents are around.”

*Passive sharing and intervention between students and teachers*: Students rarely inform their teachers about their health problems or seek advice from them, but teachers make little effort to prevent such problems (such as evaluating health problems, providing education, etc.).

“I don’t think I had such a conversation with my teacher.”

##### Consequences

*Forming diverse digital habits*: Middle school students formed their own habits of using smart devices even at home in accordance with contracts and regulations. In addition, some students were found to have replaced their mobile phone with a phone without any smart functions. On the other hand, without having positive habits for the use of smart devices, students will keep using them, even at night when they are lying in bed with the lights off. Due to a lack of sleep, students who did this experienced repeated fatigue and interference with their studies. Further on, some students even reported the increased use of smart devices for purposes other than learning when their parents went out or on holidays.

“When I swapped my cellphone for one that doesn’t have smart features, my body and mind felt better. If the time I spent on a cellphone equaled 100% when I had a smartphone, it has now been reduced to about 1%. The best thing about it is that I no longer hear mom saying I’m stuck on the phone all the time.”

*Differences in ability to control health problems*: Since students noticed that smart learning habits could cause eye and musculoskeletal symptoms, they had to decide between desirable and undesirable ways to prevent these problems.

“After looking at the tablet screen for a while, I get this unusual burning sensation in my eyes. When this happens, I stop looking at the computer screen and get up […] then I take a break, trying to look at something else other than the computer screen. I look at far-away buildings.”

In summary, this category relates to middle school students using strategies that can improve their self-control in the use of smart devices to prevent health problems. The positive results from such efforts can make students form healthy digital habits to prevent health problems, such as keeping a close watch on themselves so as not to deteriorate; however, there were also some instances where students neglected their health problems because they overlooked the importance of good usage habits and the serious problems they could experience.

#### 3.2.2. Process Analysis

Process analysis is the sequential analysis of the actions and interactions involved in the response, coping, and regulation of central phenomena that change over time [29]. In this study, the central phenomena, actions–interactions, and the results derived from paradigm analysis are represented as cyclical processes over time to identify the process of middle school students coping with health problems in smart learning. As a result, students were able to see for themselves that smart learning not only creates health problems but also creates desirable digital habits through interactions with their parents. On the contrary, students only noticed misinformed knowledge and impatience forming ineffective digital habits.

### 3.3. Selective Coding

#### 3.3.1. Core Category: Forming of Ideal Digital Learning Habits

After analyzing the data from this study, the core category was “forming ideal digital learning habits.” Middle school students experienced physical and psychological health problems while using smart devices in their learning activities. The students tried to solve their own health problems by searching for health information or by requesting help from their parents and from the school system. In the interactions between students and parents, the rules for using smart devices were set and dealt with informally; however, while there were positive aspects of how students themselves cope with health problems, there were also adverse physical and psychological aspects. Regarding the physical aspects, misinformed knowledge caused more health problems, while regarding the psychological aspects, new dysfunctional effects such as impatience appeared. As such, the way students cope with health problems related to smart learning was being formed in various ways that were either effective or ineffective.

#### 3.3.2. Generating Hypotheses and Theory

The hypothesis of this work established a hypothetical formalization through core categories and contextual conditions, and then constructs hypothetical relationship statements through action–interaction strategies.

Contextual conditions:

- High dependence on smart devices and high anxiety over uncertain information;
- Low dependence on smart devices and high anxiety over uncertain information;
- High dependence on smart devices and low anxiety over uncertain information;
- Low dependence on smart devices and low anxiety over uncertain information.

Core category: Forming ideal digital learning habits.

**Hypotheses** **(H1).** *The higher the dependence on smart devices, the more students will form ineffective digital learning habits.*

**Hypotheses** **(H2).** *The more students rely on uncertain information, the more they will form ineffective digital learning habits.*

**Hypotheses** **(H3).** *The lower the dependence on smart devices, the more students will form effective digital learning habits.*

**Hypotheses** **(H4).** *The lower the dependence on uncertain information, the more students will form effective digital learning habits.*

**Figure 2:**
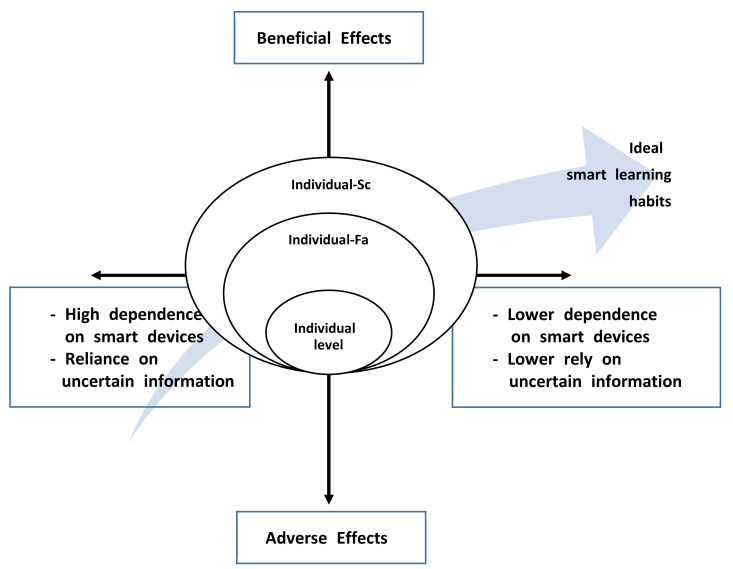
A model for ideal digital habits with the use of smart devices.

Researchers presented the conditional matrix of “forming ideal digital learning habits” from 3 levels in Figure 2 (individual level, individual–family level, and the individual–school level). The conditional matrix is a step towards summarizing and integrating results from this research process. The various situational conditions associated with the phenomenon relating the actions and interactions of micro- and macroscopic conditions are explained by integrating them into a final step to show how they affect the results from all categories.

The theory of “the process of forming ideal digital learning habits” for health problems related to smart learning for students is as follows. The process of forming an ideal digital learning habit includes beneficial effects, adverse effects, and potential adverse effects.

Beneficial effects occur under conditions of low dependence on smart devices and low anxiety over uncertain information. At this stage, students actively utilize their own coping skills, their parents, and school systems. Through this, effective digital learning habits are formed.

Adverse effects occur under conditions of high dependence on smart devices and high anxiety over uncertain information. At this stage, students create dysfunctional digital learning habits by increasing psychological instability, such as false knowledge and impatience.

Potential adverse effects occur under conditions of low dependence on smart devices and high anxiety over uncertain information and high dependence on smart devices and low anxiety over uncertain information. At this stage, students progress to a dysfunctional stage if they do not manage any psychological anxiety, due to potential adverse factors such as false knowledge or impatience.

## 4. Discussion

We examined middle school students regarding their experiences related to health problems when they use smart devices for learning. As a result, what we call a “health problem experience” is shown to be the central phenomena of smart learning. The derived health problems were classified into six categories: visual, musculoskeletal system, auditory symptoms, systemic symptoms, passive attitude, and decreased concentration. These kinds of health problems associated with long-term computer work are similar to the subjective symptoms of VDT syndrome. Previous studies have also described students’ discomfort when they use computers, and their coping strategies [14,15,16,19,31,32,33].

Regarding major health problems related to using computers and smartphones, symptoms related to the eyes were examined first. According to our results, during smart learning, dry eye syndrome can occur faster and be worse when students use contact lenses for appearance. This is a similar result to a report by Keating (2015), who noted severe dry eye symptoms (including VDT-related symptoms when wearing contact lenses, etc.) [34]; therefore, it can be said that the actions students take to correctly recognize and practice healthy habits appear to affect their coping strategies during smart learning.

Second, frequent musculoskeletal symptoms such as lower back pain [35] were reported, while a way to alleviate this problem by using chairs with back support was found; however, musculoskeletal symptoms could also be related to monitor height [32]. A method for calibrating this has not yet been investigated, which shows that further investigation is needed before intervention programs for this problem can be planned. In short, to prevent student musculoskeletal symptoms, it is necessary to create an appropriate environment for smart learning that includes the use of aids.

Hearing problems caused by using earphones were also reported. Smart devices have the advantage of unique mobility [34], which allows students to learn even while walking or on public transportation; however, this means that students use earphones due to ambient noise. Students complained about becoming temporarily deaf and hearing soft sounds from afar due to hours of earphone use at high volume. In one study, loud sounds such as music through earphones and portable audio devices caused temporary deafness and even strange sounds, but this kind of result is hard to find in the existing literature. Such findings partly replicate the symptoms mentioned by participants in a study about damage to the ears when earphones are removed after listening to loud sounds [36]. Another study on the ability of elementary school students to use smartphones also reported that they frequently use earphones [37]. Using earphones (or headphones) while walking or using public transportation could cause major accidents, so they should not be used when traveling [33,38,39]. There is an urgent need to investigate new auditory symptoms that arise from using smart devices and to make plans to monitor and assess the number of safety accidents due to the mobility of smart devices. Furthermore, intervention plans to promote the safest and healthiest ways to use the mobility functions of smart devices should be put into place for school-age children.

Strategies for alleviating students’ health problems include self-led coping, coping through mutual contracts between them and their parents, and passive sharing and intervention between them and their teachers. Students tend to neglect the emotions they feel during smart learning because they believe the feelings are insignificant, since they do not feel any physical discomfort. This kind of neglect was noticeable for the family coping strategies of limited blame or unconditional forced limitation on usage. According to a qualitative study based on the experience of 12-year-olds using smartphones, students disagree with their parents’ unconditional ban on smart devices and their concerns about studying with smartphones. [33]. Some choose to refrain from using their smartphones excessively according to their parents’ wishes, but most students ignore parental control and increase their use. A model including alternatives should be arranged at home, because our study found that students did not practice coping behaviors to express their bad feelings or ask their parents for help. Furthermore, the experiences of students have shown that even when smart devices are used in education, schools do not manage their use [40]. Students began to use smart devices for learning before teachers had the chance to associate them with education; thus, they lacked the ability to cope with this kind of trend.

As a result of selection coding, the core category is “forming ideal digital learning habits”. The hypothesis of this study was that “students will form variable digital learning habits through action–interaction”. Through this, “types of hypotheses” were created as a “beneficial effect”, “adverse effect”, and “potential adverse effect”.

Beneficial effects arise under conditions of low dependence on smart devices and low anxiety over uncertain information, in which students actively seek information for themselves to solve problems or even seek help from friends and parents or from school teachers.

Adverse effects arise under conditions of high dependence on smart devices and high anxiety over uncertain information, whereby students rely on false knowledge; they do not receive help from friends or parents and are passive in student–teacher relationships.

Potential adverse effects arise under conditions of low dependence on smart devices and high anxiety over uncertain information and with high dependence on smart devices and low anxiety over uncertain information, because the most important part of this step can have beneficial effects if it is well-controlled.

The era of digital learning will accelerate in the future, and the use of smart devices is inextricably linked, so measures have to be taken to ensure that students use smart devices in the most ideal way.

## 5. Conclusions

This study analyzed health-related issues in the digital era, in which smart devices are used by middle school students, using grounded theory. Adverse health effects in terms of unsafe smart learning behavior and inefficient smart learning environments are indicated as part of a central phenomenon of experience with health problems, which are caused by a high dependency on smart devices, an unhealthy lifestyle, and anxiety due to uncertain information. Our study shows that systemic symptoms (eyes, ears, musculoskeletal system, etc.) and psychosocial symptoms in the form of passive attitude and decreased concentration occur.

These phenomena are mediated through the student’s coping competence, the family’s coping pattern, and the school support system, which were derived in terms of two situations of positivity and negativity through the relationships between students and teachers. This indicates that students form digital habits such as ambivalent use of smart devices and have differences in their ability to control health problems. Since the use of digital devices cannot be avoided, we endeavored to present best practices for the use of digital devices. With the ideal digital learning habits presented in this study, students can develop their intrapersonal skills and try to cope with a positive attitude while using smart learning to study or to handle their health problems and ask for help as a first step.

The core category of this study was “forming ideal digital learning habits”, which formalized the hypothesis through the core categories and contextual conditions and constructed statements of hypothetical relationships through action–interaction strategies. This shows that the process of forming an ideal digital learning habit has beneficial, adverse, and potential adverse effects.

The limitation of this research was that data were collected from four middle schools in Seoul, meaning it did not address regional differences among a more diverse group of subjects. Further studies are needed to reduce the differences in target samples. Moreover, since all of the interviewees were teenagers, they could not report their health problems as precisely as was hoped and could not present enough opinions on the subject. As the main focus of this study was to look for adverse health-related effects from digital learning, it failed to explain the other educational benefits of digital leaning. Moreover, since our data were time-sensitive, there was a limit on interpretation at this point. Nevertheless, analyzing the actual experiences of students using smart devices in the era of digital learning can be considered meaningful, as it will be the basis for further important empirical research.

## Figures and Tables

**Table 1:** In-depth interview questionnaire.

Stage	Interview Topic	Sample Questions
Introductory questions	Use experience and frequency	Have you ever used a computer or a smartphone for learning? If so, how many times a week do you use it?
Transition questions	Attitude about usage	Do you think learning from smart devices is more useful than learning from books? Or do you think it is disturbing?
Key questions	Status issues with using smart devices	Have you ever experienced any kind of health-related inconvenience or concern over the past year while using smart devices for learning?
	Coping with health problems	If so, how did you deal with it? How did you deal with any inconvenience or problems with your smart device while or after you used it?
	Factors affecting health problems and results after coping	In what circumstances do you feel the discomfort or feeling is getting worse? How do you think it will help you when it comes to dealing with an uncomfortable situation?
	Health support system	Who did you ask for help when you felt uncomfortable? What kind of help did they offer you?
Final questions	Summary and additional comments	Is the content summary to this point appropriate?
		We have been talking about the health problems of smart learning so far. Do you have anything else that you want to add?

**Table 2:** Classification of identified health issues related to smart learning using the grounded theory approach.

Paradigm	Categories	Subcategories		Concepts
Causal conditions	Unsafe smart learning behavior	Learning without a break for a long time		Watching lectures for longer than 2 h on a regular basis
		Danger when using a smartphone while walking		Danger (standing on bus, subway, etc.) when watching lectures on smartphone non-stop while walking
		Unhealthy habits and posture when using a smartphone		Due to small letters on smartphones, the distance between one’s eyes and the device is closer than the distance when reading a textbook Posture affected when laying down a smartphone to watch in a lying position
	Inefficient smart learning environment	Inefficient use of support devices		Engaging in smart learning when sitting on a chair without a backrest or a monitor stand
		Smart device malfunction occurred		If the computer breaks down while working, it is very irritating and can affect one’s concentration
Context	High dependency on smart devices	Use of smart devices indiscriminately for long periods of time for purposes other than learning		Greatly increased non-learning activities (Internet browsing, SNS, etc.) Too much dependency on SNS; indiscriminate use when parents are not present
	Unhealthy lifestyle (daily)	Wearing circle lenses for aesthetics, using earphones for long periods of time		Wearing circle lenses causes dry eye syndrome Reduced hearing due to extended earphone use
	Anxiety due to uncertain information	Vague anxiety due to unconfirmed information		Fear of negative effects of electromagnetic waves on brain development and health
Central phenomena	Experience with health problems	Experience of physical health problems	Eye symptoms	As the eyes become easily tired, vision will degrade, eyes will feel heavy, stuffy, dry, and irritated
			Musculoskeletal symptoms	Discomfort and pain in neck, shoulders, back, and wrists
			Hearing symptoms	Feeling of deafness is uncomfortable; hearing loss
			Systemic symptoms	Feeling sleepy, blurry, tired, and dizzy at the same time
		Experience of psychosocial health problems	Passive attitude	Not trying to understand the content after engaging in smart learning but studying in a passive posture and blankly learning the content due to a lack of interest
			Decreased concentration	When studying with digital tools, cannot resist the temptation to consume entertainment (SNS, Internet surfing, games, etc.), unlike with normal textbooks, so concentration is always poor
Intervening situations	Student’s coping competence	Searching for health knowledge with digital information		Searching the Internet for similar answers, searching for ways to reduce problems and get similar information about others’ experiences, and figuring out how to respond with their own strategies
		Trying to maintain a positive attitude while using smart learning		Raising interest in smart learning through curiosity and satisfaction with various types of information, convenience, etc.; being able to learn on mobile device
		Ability to handle health problems and ask for help		To properly deal with health problems, regular rest and exercise are needed, along with asking for help immediately whenever any symptom of discomfort appears
	Family’s copingpattern	Friendly support		Parents approve the use of smart devices only for learning purposes, and in exchange they will make time to listen to their children’s situations seriously; they will try to decide how to prevent problems if they have trouble together as a family
		Opposition		Forcing students to reduce their use of smart devices by endlessly talking negatively about them
	School (friend, teacher) support system	Sharing empathy for uncomfortable situations with friends		Sharing sympathy about an uncomfortable situation, and consoling each other when experiencing difficult feelings
		Teachers convey and support relevant information		School provides videos on ways to manage health problems During class, teacher will explain the causes of health problems from the use of smart devices Students can learn from compulsory education provided by schools to deal with health problems when they appear Students can practice with their teachers how to prevent health problems at school
Action–interaction strategies	Student’s self-led coping	Does not express discomfort to anyone		Students do not express feelings of discomfort to parents, siblings, friends, or teachers, or ask people around them for advice or help on ways to reduce such feelings
		Endures every uncomfortable feeling unconditionally		Students ignore feelings of discomfort and endure them alone, thinking they will be fine if they take a short break, but when it is too hard to bear, they stop learning
		Takes a rest when feeling uncomfortable		When feeling uncomfortable (eye fatigue, low back pain, numbness, etc.), student immediately takes a break
		Practices action and response methods to mitigate discomfort		Students try to repeat basic exercises they already know (stretching, changing posture, blinking their eyes, etc.), smart devices will also allow them to rest their eyes for a short time; if they have eye drops, they can put them in for hydration
		Telling parents about feelings of discomfort and trying to improve the problem		Expressing discomfort to family (especially the mother) based on the environment and try to follow the family’s guidance
	Coping through mutual contracts between students and parents	Setting rules for using smart devices between parents and students		Discussing use of smart device, setting up and following rules (use time, work, circumstances, etc.) with parents, and remembering to follow the rules as promised
		Using materials with parents to help prevent health problems		When using a computer at home, parents prevent back pain by using a chair with a backrest and props to adjust monitor height to eye level
	Passive sharing and intervention between students and teachers	Passive health problems arise between teachers and students		Without informing teachers about students’ health problems, opportunities for counseling on smart learning and health problems are not available, but teachers still will not do anything to prevent them
		Teachers cannot actively intervene		There are no exact guidelines on adverse effects when using smart devices, not all teachers are accustomed to using them, and not many teachers are aware of the side effects of using smart devices at school
Consequences	Forming diverse digital habits	Ambivalent use of smart devices		Students cannot resist playing with their smart devices when their parents are absent or on holidays; most of them turn off the lights and sneak it in their beds and play non-stop
				Some students try to overcome the temptation to use their phones too much and exercise self-control by replacing their smartphone with a 2G phone that only allows conversation and text messaging
		Differences in ability to control health problems		Health issues due to smart learning are a necessary evil, so students should be patient and stop using smart devices temporarily to address their health problems
				Active students monitor their own health problems and attempt to properly deal with them and try to prevent them

## Data Availability

Not applicable (This is secondary data).
